# Genetic loci inherited from hens lacking maternal behaviour both inhibit and paradoxically promote this behaviour

**DOI:** 10.1186/s12711-015-0180-y

**Published:** 2015-12-30

**Authors:** Atia Basheer, Chris S. Haley, Andy Law, Dawn Windsor, David Morrice, Richard Talbot, Peter W. Wilson, Peter J. Sharp, Ian C. Dunn

**Affiliations:** Roslin Institute and Royal (Dick) School of Veterinary Studies, University of Edinburgh Easter Bush, Midlothian, EH25 9RG Scotland, UK; Animal Breeding and Genetics Section, Department of Livestock Production, University of Veterinary and Animal Sciences, Ravi campus, Lahore, Pakistan; Edinburgh Genomics, Ashworth Laboratories, The University of Edinburgh, Edinburgh, EH9 3JT UK

## Abstract

**Background:**

A major step towards the success of chickens as a domesticated species was the separation between maternal care and reproduction. Artificial incubation replaced the natural maternal behaviour of incubation and, thus, in certain breeds, it became possible to breed chickens with persistent egg production and no incubation behaviour; a typical example is the White Leghorn strain. Conversely, some strains, such as the Silkie breed, are prized for their maternal behaviour and their willingness to incubate eggs. This is often colloquially known as broodiness.

**Results:**

Using an F_2_ linkage mapping approach and a cross between White Leghorn and Silkie chicken breeds, we have mapped, for the first time, genetic loci that affect maternal behaviour on chromosomes 1, 5, 8, 13, 18 and 19 and linkage group E22C19W28. Paradoxically, heterozygous and White Leghorn homozygous genotypes were associated with an increased incidence of incubation behaviour, which exceeded that of the Silkie homozygotes for most loci. In such cases, it is likely that the loci involved are associated with increased egg production. Increased egg production increases the probability of incubation behaviour occurring because egg laying must precede incubation. For the loci on chromosomes 8 and 1, alleles from the Silkie breed promote incubation behaviour and influence maternal behaviour (these explain 12 and 26 % of the phenotypic difference between the two founder breeds, respectively).

**Conclusions:**

The over-dominant locus on chromosome 5 coincides with the strongest selective sweep reported in chickens and together with the loci on chromosomes 1 and 8, they include genes of the thyrotrophic axis. This suggests that thyroid hormones may play a critical role in the loss of incubation behaviour and the improved egg laying behaviour of the White Leghorn breed. Our findings support the view that loss of maternal incubation behaviour in the White Leghorn breed is the result of selection for fertility and egg laying persistency and against maternal incubation behaviour.

## Background

In birds, incubation behaviour, often colloquially known as broodiness, is a facet of maternal behaviour and is a complex trait. It is the act of sitting on a nest of eggs to incubate them and precedes brooding, which begins when the chicks hatch. Thus, it is more correct to consider incubation behaviour as the trait of sitting on eggs and we have used this term throughout this paper to distinguish it from the care of the hatched chick, although it is also frequently referred to as broodiness. Incubation behaviour results from the interaction between the hormonal system and the environment of the bird [1] and is characterised by persistent nesting, turning and retrieval of eggs, clucking and nest defence. It is associated with increased secretion of prolactin and decreased secretion of luteinising hormone and subsequent regression of ovaries and oviduct, and cessation of egg production [1–4]. A combination of the correct hormonal milieu and suitable environmental conditions such as high temperature, presence of eggs and nest are key factors which encourage incubation behaviour [1, 5]. This behaviour is inhibited if eggs are removed from the nest as they are laid or roll away from the nest in the cage [6].

During domestication, incubation behaviour has been lost in some breeds of chicken, notably the White Leghorn (WL) breed [5], while many breeds of poultry including Red Jungle fowl [7] have retained this behaviour with hens incubating their own eggs. The Egyptians followed by the Chinese perfected artificial incubation methods on a large scale at least 3000 years ago [8]. We believe that this revolutionary technology was a pre-requisite for the development of breeds of chickens in which incubation behaviour does not occur and for the disruption of the link between maternal behaviour and reproduction. Because incubation behaviour is associated with a cessation of reproduction during many weeks, there was strong motivation for breeders to develop breeds that have lost this trait to produce highly productive egg laying strains. The original jungle fowl lays 10 to 15 eggs per year in the wild whereas modern strains produce 300 eggs per year [9], which would be impossible if they showed any signs of incubation behaviour. Indeed, selection for the absence of incubation behaviour can be considered as selection for a very long clutch of eggs.

The WL breed belongs to the Mediterranean class of chicken breeds. It has a high egg production rate and incubation behaviour in this breed is practically absent [5]. It has been suggested that this trait is under the control of a major gene [1, 4, 10]. The Silkie (SLK) breed, which is bred mainly for ornamental purposes, carries alleles for many Mendelian traits including polydactyly (Po), silkie feathering (h), fibromelanosis (Fm) and rose comb (R) [5] and has a high incidence of incubation behaviour [11]. SLK belongs to the Asiatic class and is thought to have originated in China [12].

Differences in the incidence of incubation behaviour between breeds and between selection experiments suggested that this trait may have a significant heritable component [5] but investigations on the genetics of incubation behaviour have led to conflicting observations. Some of the earliest studies [13] showed that incubation behaviour is controlled by more than one independent autosomal gene and a within-family approach on Rhode Island Red hens supported the autosomal basis for this trait [14]. Reports suggesting that this trait was controlled, at least to a large extent, by sex-linked genes [9, 15] contradicted these earlier findings. Later, the hypothesis that sex-linked genes were responsible for incubation behaviour [15] was disproved by Romanov [4] who analysed reciprocal and F_1_ back crosses between WL and Bantam chickens. Overall, it is generally accepted that more than one locus contributes to the absence of incubation behaviour in strains like the WL.

Determining the molecular mechanisms that underlie incubation behaviour is expected to help increase egg production by selection for or introgression of the underlying loci into breeds of hens that are adapted to local conditions and favoured by rural farmers. Such local breeds often have low productivity due to the onset of incubation behaviour. In developing countries, traditional village-scavenging poultry make up a significant proportion of the national flock [16]. Use of modern molecular genetics techniques and resource populations to detect quantitative trait loci (QTL) for incubation behaviour offers the opportunity to identify candidate genes that control maternal behaviour and explain its molecular basis. In addition, identification of the loci that were selected for, or against, during the process by which this trait was lost in breeds like the WL will give us an insight into the processes of domestication of chicken and how this selection has evolved with artificial incubation.

## Methods

### Resource population

**Fig. 1:**
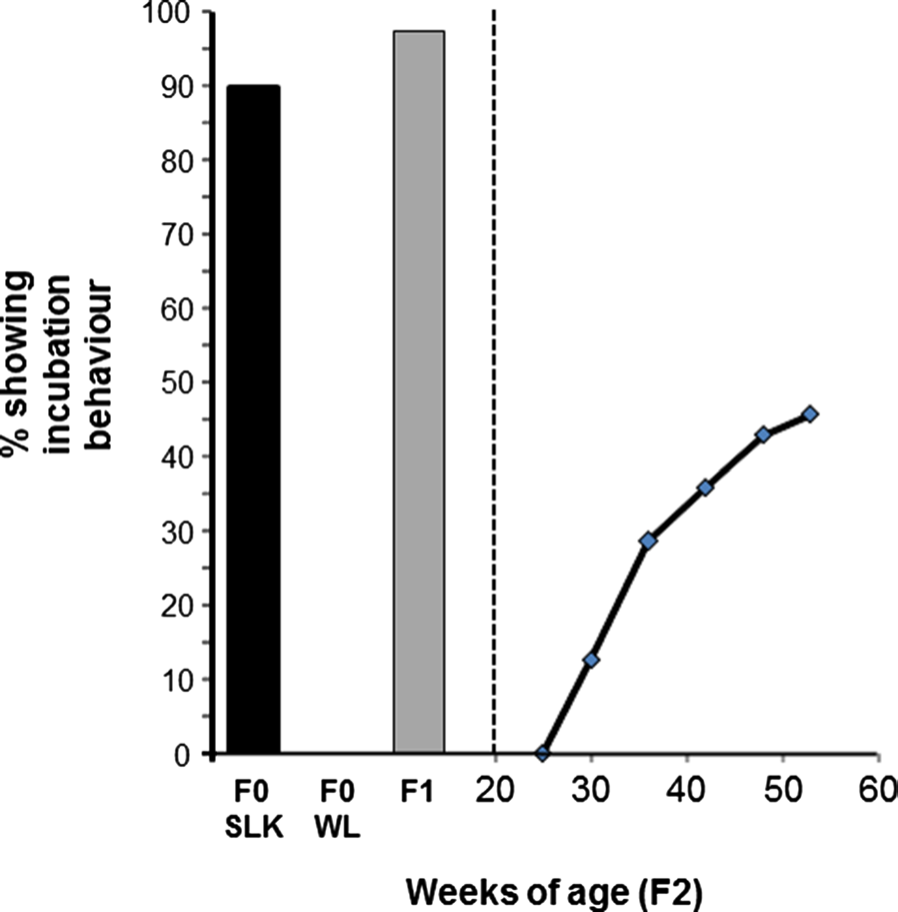
Percentage of hens that show complete incubation behaviour (incubation status trait class 1) in the founder SLK and WL breeds (F_0_) and in the F_1_ SLK × WL cross represented as a *bar graph* on the *left hand* of the graphic. On the *right hand* side of the graphic the cumulative incidence of incubation behaviour over the period of testing in the F_2_ population is represented as a line graph with the x-axis indicating weeks of age. At week 50 this is equivalent to the trait ‘incubation status’ class 1. For all data the y-axis represents the percentage of hens showing incubation behaviour (incubation status trait class 1)

Two divergent breeds of chicken, WL and SLK were used to set up the F_2_ cross used in this study. WL chickens were from a flock that is maintained at the Roslin Institute and showed no incubation behaviour (0 % incidence) when tested by the same method as that used to record phenotypes for the F_2_ population (Fig. 1). SLK chickens were obtained from the Wernlas Collection (Shropshire, SY7 9BL), a certified rare breeds farm (now closed), and maintained at the Roslin Institute. In this breed, incidence of incubation behaviour reached 90.5 % when tested (Fig. 1). All matings were performed by artificial insemination. Three WL sires were crossed with eight SLK dams and two SLK sires were crossed with ten WL dams in the F_0_ generation to produce the F_1_ cross. Four males and 20 females from the F_1_ generation were used to establish the F_2_ population. Phenotypic data for incubation behaviour were successfully recorded on 280 F_2_ animals from 19 families. This population was already used in a study that determined the causative mutation for preaxial polydactyly [17]. All animal experiments were performed according to United Kingdom Home Office legislation and were approved by the ethics review group of the Roslin Institute.

### Incubation behaviour phenotype

After hatching, chickens of the F_1_ and F_2_ generations were reared in floor pens on a short-day lighting schedule (8 h light and 16 h dark) for 16 weeks. After 16 weeks, the birds were transferred to new floor pens (4 m × 1 m) in groups of six or seven individuals on a long-day lighting schedule (16 h light and 8 h dark). Temperature was maintained between 18 and 23 °C and the birds had access to food and water ad libitum. Each pen contained nest boxes, with wood shavings and hard-boiled eggs to encourage incubation behavior. Daily behavioral observations recorded birds that displayed persistent nesting (i.e. not leaving the nest if challenged), raising feathers and clucking when approached. Birds for which this behavior persisted for two consecutive days were referred to as incubating. Freshly laid eggs were removed from the pens but not the hard-boiled eggs. The birds were maintained in these conditions up to the age of 1 year. The number of days between entering the pen and the onset of incubation behaviour was also recorded.

### Treatment of the data for analysis

Data for analysis were prepared in two ways. First, for the trait termed ‘incubation status’, the data were categorised on a 3-point scale i.e. there were three categories of birds: category 1 included birds that clearly showed incubation behaviour including vocalisation, raising feathers when approached and sitting on the eggs; category 2 included birds that showed some signs of incubation behaviour, for example, vocalisation and/or raising feathers when approached, but no persistent sitting; and category 3 included birds that showed no signs of incubation behaviour. In the second approach, the data were split into cumulative time periods because we believed that the time needed for hens to start incubation behaviour might reveal information on the strength of the motivation for incubation. The experiment was split into five cumulative time periods, by categorising and analysing birds that showed full signs of incubation behaviour from 25 to 30, 25 to 36, 25 to 42, 25 to 48 and 25 to 53 weeks of age. However, since significant or suggestive results were only obtained for the first period, in this paper, we consider only the first period between 25 and 30 weeks of age, which is referred to as ‘early incubation behaviour’. The phenotyping data were considered as a bivariate trait with two classes i.e.: class 1, which included birds that did not display any incubation behaviour, and class 2, which included birds that did have incubation behaviour (See Additional file ‘Phenotype’ accessible at Dryad Digital Repository).

### Genotyping

Blood samples were collected from all F_0_, F_1_ and F_2_ individuals and DNA was extracted as previously described [18].

### Microsatellite markers

Based on an initial screen, 90 microsatellite markers that were known to be informative for these populations and to be spread across 23 autosomal linkage groups and the sex chromosomes were used to genotype F_0_, F_1_ and F_2_ animals (See Additional file ‘Genetic Map Distances’ and Additional file ‘Genotypes’ accessible at Dryad Digital Repository). Fragment sizes were determined using GENESCAN 3.1 DNA fragment analysis and GENOTYPER 2.1 (PE Biosystems, Foster City, USA).

### Single nucleotide polymorphisms (SNPs)

After an initial analysis for potential QTL, the number of markers on chromosomes 2, 5, 7, 9, 13, Z and linkage group E22C19W28 which had putative QTL was increased and linkage groups which were not represented in the initial microsatellite screen were added (19, 20, 21, 22, 24, 25, LGE64) by genotyping the entire population for 384 SNPs. SNPs were selected from the Ensembl genome browser (http://www.ensembl.org/Gallus_gallus/Info/Index) and a list of published validated SNPs [19]. SNP genotyping was done using the GoldenGate genotyping assay with VeraCode technology (Illumina) and Bead Studio software was used to analyse SNP data and, as a first quality control, to remove poor quality or uninformative SNPs. Of the 384 SNPs, 218 were informative and of suitable quality. After these 218 SNPs were added to the map, a further 32 SNPs were added in the peak region of the best QTL on chromosome 5, some of these SNPs being located in coding regions. Genotyping was also carried out using the GoldenGate genotyping assay with VeraCode technology (Illumina). These SNPs were selected by comparing the whole-genome sequences of the SLK and WL breeds, which were obtained by Illumina^®^/Solexa next-generation sequencing. (See Additional file ‘Genetic Map Distances’ and Additional file ‘Genotypes’ accessible at Dryad Digital Repository).

### Software used

All pedigree information, marker genotypes and phenotypic data were stored in the ResSpecies database [20]. Each marker was checked for individual and pedigree errors using the related ResSpeciesGenotypeChecker (http://resspecies.org) software prior to submission and poor quality data were removed [21].

### Map construction

For map construction, both microsatellites and SNPs were exported from ResSpecies in the CRIMAP format. Marker order and map distances were estimated by using CRIMAP 2.4 software (http://saf.bio.caltech.edu/saf_manual/crimap-doc.html). For chromosomes 5 and 8 that contained many SNPs, the size of the maps was very large. Thus, we used the CRIMAP CHROMPIC option to identify unlikely double crossovers, which reduced the map of chromosome 5 to a more realistic length. For chromosome 8, it was necessary to construct a map by interpolation between the microsatellite genetic map and the SNP physical map that was obtained by using the 2006 genome build. The CHROMPIC option FLIPS was used with a 4-marker window to obtain the most likely order. A sex-average linkage map was built by using the BUILD option. All markers used in this study were referenced in the chicken genome database (http://www.genome.uc-sc.edu/) to obtain their positions on the genome. The linkage map used in the analysis is in Additional file ‘Genetic Map Distances’ accessible at Dryad Digital Repository). For the comparison between the genetic and physical maps, all positions were converted to the 2011 build of the chicken genome (galGAL4) using the liftOver tool (http://genome.ucsc.edu/util.html).

### QTL mapping

Genotypic and phenotypic data for each trait were exported from the ResSpecies database. The interval mapping method [22] for QTL analysis was carried out using GridQTL [23] which is a grid-based portal version of the QTL Express program [24]. A linear model for the additive and dominance effects of a QTL at a given position was analysed by least squares for each trait with the additive effect defined as half the difference in the mean phenotypes between the two homozygotes and the dominance effect as the difference between the mean phenotype of the heterozygote and the average mean of the homozygotes. Family, pen and pen year were included as fixed effects in the model for ‘incubation status’ and for each of the cumulative periods. F-statistic profiles were generated at 1-cM intervals. We analysed the data by assuming that there were one or two QTL on each chromosome. The significant QTL that were detected were added as cofactors in the analysis of the remaining chromosomes in order to reduce background noise and increase the power of the analysis to identify additional QTL.

To give a realistic estimate of the proportion of the total phenotypic difference between breeds explained by a QTL, we estimated the mean phenotypic values for each genotype at the nearest marker to the QTL position that was fixed for the alternative QTL alleles between the SLK and WL breeds. The values for the trait were calculated for the WL–WL, SLK-WL and SLK–SLK allele combinations, and the size of the QTL effect was estimated as the proportion of the total phenotypic difference between breeds explained by the QTL expressed as a percentage. Thus, the QTL effect at the marker was twice the estimated additive effect divided by the phenotypic difference between the founder breeds for each trait. This is referred to as the ‘phenotypic difference’ to distinguish it from the ‘phenotypic variance’.

### Determination of significance thresholds

In single- and two-QTL analyses, significance thresholds were determined by conducting 5000 permutations [25] and 1000 bootstraps were used to generate 95 % confidence intervals [26, 27]. A QTL was considered as significant if its F value was greater than the P ≤ 0.05 experiment-wide threshold value [28] and as suggestive if it exceeded the P ≤ 0.05 chromosome-wide threshold. With 23 independent autosomes analysed, this is approximately equivalent to the expectation of 23 × 0.05 = 1.15 false positive QTL per genome scan.

## Results

### Summary statistics

The F_0_ SLK and WL hens tested in the same environment had an incidence of incubation behaviour of 90.5 and 0 %, respectively. Tested in the same environment, the F_1_ SLK × WL hens had an incidence of recorded incubation behaviour of 97 %, which was the highest incidence observed in this study (Fig. 1).

Test statistics for incubation behaviour showed that 126 (46 %) of the 276 F_2_ hens displayed complete incubation behaviour at the end of the test period at 50 weeks of age (Fig. 1), 77 (28 %) showed partial incubation behaviour and 72 (26 %) birds showed no sign of incubation behaviour. The onset of incubation behaviour varied between 61 and 140 days for the birds that had incubation behaviour. These data on the development of incubation behaviour in the cumulative time periods are in Fig. 1 alongside data for ‘incubation status’ in the F_0_ and F_1_ hens for comparison.

### Incubation status

For ‘incubation status’, the threshold value for an experiment-wide QTL (P ≤ 0.05) was F = 8.1 and the chromosome-wide significances for chromosomes 1, 5, 18 and linkage group E22C19W28 were F = 7.5, 7.7, 5.3, and 5.6 at P ≤ 0.01, respectively and F = 5.6, 5.8, 3.6, and 4.2 at P ≤ 0.05, respectively. Thus, there was evidence (F = 7.84) for a chromosome-wide significant QTL on chromosome 5 at 100 cM (Table 2) and three other suggestive QTL for ‘incubation status’ were detected after adding SNPs on chromosome 1 at 70 cM, chromosome 18 at 0 cM and in the linkage group E22C19W28 at 13 cM with F-values of 5.34, 5.51 and 6.01, respectively. Another suggestive QTL for ‘incubation status’ on chromosome 19 at 1 cM was identified when the four QTL on chromosomes 5, 1 and 18 and in linkage group E22C19W28 were fitted as background co-factors (Table 2).

After adding 218 informative SNPs across the genome, the 95 % confidence interval (CI) of the QTL on chromosome 5 spanned a region of about 48 cM (Table 2) instead of the 95 cM region previously identified. The peak position of the QTL on chromosome 5 was initially flanked by the microsatellite markers LEI0145 and MCW0032 and, after adding SNPs, it was reduced to a region between SNP rs13587819 and MCW0032. Thirty-two additional SNPs were genotyped in the region between rs13587819 and MCW0032 and four line-specific markers were identified, three in the region that contains the *DIO2* (*deiodinase, iodothyronine, type II*) gene and one in the *TSHR* (*thyroid stimulating hormone receptor*) gene. After analysis of these line-specific markers, the peak position of the QTL was found to be close to the *TSHR* SNP and its physical position on chromosome 5 was around 40,087,100 bp (galGal4 assembly of the chicken genome).

All the QTL detected for ‘incubation status’ had dominance effects and the two QTL on chromosomes 5 and 19, respectively, were clearly over-dominant (Table 3). For example, the dominance effect of the QTL on chromosome 5 was equal to 0.51 (Table 3). The scores for incubation status ranged from 1 (complete incubation) to 3 (no incubation), which means that the standardized dominance effects explained 12.03 % of the trait standard deviation and 8.97 % of the phenotypic variance. However, it is more meaningful and allows to interpret results more easily if the informative marker that is nearest to the QTL peak is considered to estimate the genotype means rather than to rely on the estimated effect. Based on the size of the effect of the QTL on chromosome 5, the heterozygous individuals were less likely to show incubation behaviour than either of the homozygous individuals (Table 4). Conversely, for the QTL on chromosome 19, its effect was reversed with heterozygous individuals more likely to show incubation behaviour (Table 4). The remaining QTL appeared classically dominant rather than over-dominant. For the QTL in linkage group E22C19W28 and on chromosome 18, the dominant SLK alleles reduced incubation behaviour (Table 4). Only the WL allele on chromosome 1 had a dominant effect that reduced the likelihood of incubation behaviour or conversely the SLK allele promoted incubation behaviour by explaining about 26 % of the phenotypic difference between the founder breeds (Table 4). When considering all reported loci together, the overall additive effect on incubation status was equivalent to 63 % of the phenotypic difference between breeds.

However, it is clear that some of the effects do not originate from the predicted breed, for example, incubation behaviour is promoted by alleles inherited from the WL breed for the QTL on chromosome 1 and therefore has a negative sign in Table 4. Thus, the more conservative approach suggests that the effects that originate from the SLK breed explain 33 % of the phenotypic difference between breeds.

### Early incubation behaviour

For early incubation behaviour (Period 1; Table 1), the experiment-wide threshold F value for a QTL (P ≤ 0.05) was equal to 9.0 and the chromosome-wide significances for chromosomes 1, 8, and 26 were F = 7.1, 7.1, and 5.8 at P < 0.01, respectively and F = 5.5, 4.9, and 3.9 at P ≤ 0.05, respectively. QTL for early incubation behaviour were detected on chromosomes 8 at 18 cM and 26 at 0 cM. Another suggestive QTL on chromosome 1 (66 cM) was identified when these two QTL were fitted as background effects. These QTL explained the phenotypic variance in the first period between 25 and 30 weeks of age and, thus, are referred to as ‘early incubation behaviour’ QTL to distinguish them from the QTL that have an influence over the whole period which are referred to as ‘incubation status’ QTL. The most significant QTL for early incubation behaviour (P < 0.05; genome-wide significant) was detected on chromosome 8 at 21 cM with an F ratio of 11.78 (Table 2), its confidence interval spanned a region between 0 and 42 cM, and it explained 13.0 % of the phenotypic variation (Table 3). Another suggestive QTL for early incubation behaviour was detected on chromosome 26 at 0 cM and after adding 218 informative SNPs on all chromosomes, its additive and dominance effects were equal to −0.11 and 0.11, respectively (Table 3). Another suggestive QTL was found on chromosome 1 at 66 cM, when both QTL for early incubation behaviour on chromosomes 8 and 26 were fitted as background effects.

**Table 1:** Summary statistics for incubation behaviour over cumulative time periods

Period	Age in weeks	Number of incubating birds	Number of non-incubating birds
1	25–30	35	241
2	25–36	79	197
3	25–42	99	177
4	25–48	118	158
5	25–53	126	150

**Table 2:** Significant QTL found for incubation status and early incubation behaviour in the WL × SLK cross

Chromosome	F-ratio^a^	Position (cM)	CI (cM)^b^	Flanking markers around peak	Genome position (Mb)^c^
Incubation status					
5	7.84	100	75–123	snp_dio2_08-snp_tshr_06	39.82–40.09
E22C19W28	6.01	13	0–26	rs16687038-rs16705784	0.41–0. 84
19	5.93	1	0–16	rs15846285-rs15050199	5.16–8.08
1	5.34	70	1–175	LEI0146-MCW0112	53.19–65.12
18	5.51	0	0–25	ADL0304	1.44
Early incubation behaviour					
8	11.78	21	0–42	rs16624982-rs16625404	5.93–6.63
26	7.46	0	0–66	ADL0330	1.31
1	6.16	66	3–100	LEI0146-MCW0112	53.19–65.12

^a^Highest F-ratio estimated from regression analysis (genome-wide)

^b^Confidence interval

^c^Position on the 2011 genome assembly (galGAL4) of the nearest flanking markers

**Table 3:** Additive and dominance effects for incubation status and early incubation behaviour in the WL × SLK F2 cross

Chromosome	Position (cM)	Additive ±SE	Dominance ±SE	Standardized additive effect^a^	Standardized dominance effect^b^	Phenotypic variance (%)^c^
Incubation status						
5	100	0.08 ± 0.01	0.51 ± 0.12	1.77	12.03	8.97
E22C19W28	13	−0.18 ± 0.09	0.46 ± 0.15	−4.06	10.28	7.02
19	1	−0.06 ± 0.01	−0.69 ± 0.20	−1.16	−14.50	6.25
1	70	0.23 ± 0.11	−0.63 ± 0.23	4.98	−13.48	6.29
18	0	−0.24 ± 0.09	0.32 ± 0.14	−5.36	7.23	6.48
Early incubation behaviour						
8	18	0.18 ± 0.04	0.10 ± 0.05	7.35	4.21	13.02
26	0	−0.11 ± 0.04	0.11 ± 0.05	−4.49	4.63	7.09
1	66	0.06 ± 0.04	−0.36 ± 0.11	2.45	−15.16	7.36

^a^Mean additive effect divided by trait standard deviation expressed as percentage

^b^Mean dominant effect divided by trait standard deviation expressed as percentage

^c^Calculated as the proportional decrease in the residual sums of squares resulting from the full model including the QTL compared to the residual sums of squares from the reduced model from which the QTL was omitted expressed as percentage

The QTL on chromosome 8 for early incubation behaviour had an additive effect and one of its alleles in the SLK breed increased the likelihood of incubation behaviour (Table 4). However, the effect of this allele was nearly dominant. Since the trait was scored 1 or 2, with 2 for incubation, the effect of this allele represented about 12 % of the phenotypic difference between the breeds and if the loci on chromosomes 26 and 1 were included, the additive effects amounted to 25 %. However, as for ‘incubation status’, effects were not always contributed by the predicted parental breed and, thus, it is probably more accurate to consider that the effects that originate from the SLK breed explain about 12 % of the phenotypic difference between breeds.

**Table 4:** Genotype means for the informative marker nearest to the estimated QTL position

Chromosome	Marker	WL–WL^a^	WL-SLK	SLK–SLK	Proportion of the phenotypic difference between the breeds explained by the QTL (%)^b^
Incubation status (scored 1–3 with 3 corresponding to no incubation)					
5	rs13586520	1.7	1.97	1.62	−4
E22C19W28	ROS0054	1.56	1.94	1.96	20
19	rs15050199	1.89	1.65	1.91	1
1	MCW0112	1.89	1.82	1.37	−26
18	ADL0304	1.739	1.91	1.98	12.05
Early incubation behaviour (scored 1 or 2, with 2 corresponding to incubation)					
8	MCW0275	1.09	1.07	1.21	12
26	ADL0330	1.15	1.05	1.08	−7.5
1	LEI0146	1.14	1.05	1.09	−5.4

^a^Genotypes were attributed using the alleles that were fixed in the founder WL and SLK breeds

^b^Difference between the homozygote means divided by the difference between the founder breeds means for each trait (2 × additive/trait breed difference)

## Discussion

In this study, we detected a significant genome-wide QTL for maternal early incubation behaviour on chromosome 8 at 21 cM and another QTL for incubation status on chromosome 5 at 100 cM that was just below the genome-wide significance threshold. In both cases, targeted SNPs were used to refine the 95 % confidence interval (CI) containing the locus. For the QTL on chromosome 5, the 95 % CI was reduced from 95 to 48 cM between rs13587819 and MCW0032. For the QTL on chromosome 8, the 95 % CI was reduced from 89.5 to 42 cM between rs13587819 and MCW0032. The remaining QTL for incubation status on chromosomes 1, 18, 19 and in linkage group E22C19W28 were suggestive. The QTL for early incubation behaviour on chromosome 8 at 21 cM was significant at the genome-wide 5 % level. Multiple peaks were observed in the 42-cM region that contained this QTL. Data for the trait were analysed using gridQTL under a one-QTL model but this did not give a clear profile and the presence of two QTL at the locus was tested under a two-QTL model but was not supported. It is still possible that several linked QTL for this trait may be present in this region.

The peak position of the QTL on chromosome 5 coincides with the location of the largest selective sweep that was reported from a comparison between domesticated poultry breeds and red jungle fowl [29]. Thus, 32 SNPs were screened in this region. Three informative SNPs in the region of the *Dio2* gene and one in the *TSHR* gene were detected. After including these four SNPs, the peak position of the QTL moved closer to the *TSHR* SNP. A mutation in the *TSHR* gene was highlighted as being a possible target of the selective sweep [29], but to date, there is no evidence on its mode of action. Although the SLK and WL breeds do not differ at this SNP (Leif Anderson, personal communication), it has been shown that this region is involved in poultry domestication and selection on this locus could have profound effects on the biology of chickens. Indeed, analysis of the whole-genome sequence of a SLK individual revealed a selective sweep at the *TSHR* locus [30]. A more recent study on different chicken breeds that included samples of ancient DNA suggested that the selective sweep in *TSHR* might be a more recent event, perhaps dating back to only 500 years ago, rather than an initial domestication event [31]. This event could coincide with the adoption of artificial incubation during the Renaissance in Europe when progress in incubation technology spread at least to Italy, France and the UK [8]. This region contains a number of genes involved in the transduction of stimulatory photoperiodic information to the reproductive system [32]. Expression of TSHR is increased in the pars tuberalis of the brain during the first long photoperiod, and expressions of DIO2 and DIO3 are stimulated and inhibited, respectively, by the activation of *TSHR*. This results in an increased level of triiodothyronine (T3) in the brain, which is postulated to produce the biological effects of increased photoperiod on reproduction. It is striking but, perhaps coincidental, that the remaining *thyroid hormone deiodinase* gene (*DIO1*) is located in the 95 % CI of the significant QTL for early incubation behaviour on chromosome 8.

This link with the thyroid hormone system is supported by the results of the comparison between the positions of the QTL detected in this study and those in the poultry QTL database [33]. The localization of the QTL on chromosome 1 coincides with that of a QTL for T3 and T4 levels and their ratio, and for IGF-I levels in a broiler-Fayoumi cross [34]. The latter QTL was also identified using high- and low-growth lines [35]. The localization of the QTL on chromosome 1 coincides also with those of a QTL for age at first egg [36, 37] and a QTL for egg number [38]. The localization of the QTL for early incubation behaviour on chromosome 8 coincides with that of a QTL for T3 and T4 levels and their ratio, and for IGF-I levels that was detected in a broiler × WL cross [34] and also with that of a QTL for egg number [39]. Finally, the localization of the QTL on chromosome 5 coincides with that of a QTL for egg production [40]. Therefore, four of the eight significant or suggestive QTL for incubation status or early incubation behaviour, i.e. two on chromosome 1, one on chromosome 5 and one on chromosome 8, have links with the thyroid hormone system. This clearly suggests that the thyroid hormone system has an important role, but it does not tell us why it should be important for incubation behaviour. In a wild species i.e. (*Sturnus vulgaris*), it was reported that thyroidectomy inhibits the response of the reproductive axis to photoperiod and results in a longer reproductively active period [41]. We can speculate that the effects of thyroid hormones on the timing and duration of reproduction may be related to selection against incubation behaviour.

In this study, all detected loci were located on autosomes, which reinforces the view that there is no evidence of any major involvement of the Z chromosome in the control of maternal behaviour in chickens contrary to the suggestion of Saeki et al. [9]. Instead the view that at least two incompletely dominant alleles of these autosomal genes are involved in the expression of the trait is supported [4]. However, Romanov [4] speculated that two loci acted on this trait i.e. one that is responsible for incubation behaviour and another that inhibits the trait. In this study, as expected, the SLK alleles at the major QTL on chromosome 8 promote early incubation. Similarly, at the QTL on chromosome 1, a SLK allele promotes incubation behaviour whereas a dominant WL allele inhibits it. Some of the other detected loci are over-dominant, as for the major QTL on chromosome 5 for which heterozygous individuals were less likely to show incubation behaviour, or the QTL on chromosome 19 for which heterozygous individuals were more likely to show incubation behaviour. For the remaining loci in linkage group E22C19W28 and on chromosome 18, the SLK allele is dominant and decreases incubation behaviour. Therefore, although there may be some truth in Romanov’s assertion, the picture is more complex.

As previously observed [42], the incidence of incubation behaviour for the F_1_ chicken from the WL × SLK cross is high and surpasses that observed for the F_0_ SLK chicken. This illustrates that maternal behaviour is dominant, as expected. Taking this behaviour into account together with the effect of the individual loci and the physiology of the trait, we can propose several hypotheses to explain our observations. We believe that the combination of the WL high egg production and the SLK propensity for maternal behaviour with its low egg production may explain the high incidence of incubation behaviour for the F_1_ WL × SLK cross. Exposure to oestrogen and progesterone from mature ovarian follicles is a prerequisite for incubation behaviour to occur [1]. Therefore, the longer a hen is in lay, the greater is the probability that the correct steroidal environment will coincide with the correct environmental stimuli to reinforce and propagate incubation behaviour. This may explain why the loci on chromosomes 19 and 18 and in E22C19W28 have WL alleles that apparently promote incubation behaviour. These loci may in fact be involved in increased or persistent egg production, but not directly in incubation behaviour. This may be semantics, since as explained, the onset of incubation behaviour is not compatible with high egg production [1]. However, it does explain why high egg production leads to an increased potential for incubation behaviour. It also explains why none of these loci are among those found for early incubation QTL. It should be noted that, in this study, it was not possible to make individual egg recordings, thus we were not able to verify this hypothesis.

The QTL for incubation status on chromosomes 5 and 19 are over-dominant. Animals that are heterozygous at the QTL on chromosome 5 are less likely to show incubation behaviour, whereas, animals that are heterozygous at the QTL on chromosome 19 are more likely to show incubation behaviour. At least two hypotheses can be put forward to explain these observations. The two breeds used in this cross, which have been separated for a long time, may have been selected for fertility in separate populations resulting in independent fixation of beneficial and dominant alleles that are not shared between them. When these breeds are combined, as in this cross, there is an additive complementation effect of the loci on the trait. Thus, at closely linked positions, one breed may have the genotype *AAbb* and the second *aaBB* (where capital letters indicate the dominant allele for an increased incidence of incubation behaviour) and hence the F_1_ cross has the genotype *AaBb* and its phenotype for the trait is more extreme than that of either parental line. For the QTL on chromosome 5, both alleles may promote persistence of egg laying and increased egg production while the opposite may be true for the QTL on chromosome 19. An alternative hypothesis would be that the QTL on chromosome 5 may be important for both egg production and fertility but also for the maintenance of incubation behaviour, possibly via common components in the basal hypothalamic TSH to T3 production pathway which includes TSHR and the deiodinase enzymes [32]. This offers an explanation of how an allele for absence of incubation behaviour, which in naturally incubating populations of hens would not be expected to propagate because it would be effectively lethal, could increase in frequency in a population.

The combination of the two alleles at the QTL on chromosome 5 may result in a larger number of laid eggs but also in the maintenance of incubation behaviour and thus the heterozygotes have an advantage. It can be assumed that if hens do not need to incubate their own eggs because of the development of artificial incubation, those that carry the allele for absence of incubation behaviour will be strongly selected for, thus removing the restriction of incubation behaviour and increasing egg laying persistence. For the WL breed, this process may have been favoured, thus driving incubation behaviour to a minimal level and egg production to a high level.

Regardless of the hypotheses, there must be a balance between the length of time during which hens lay eggs and the termination of egg-laying by incubation behaviour, which has major implications for clutch survival in wild and domesticated species.

## Conclusions

We describe for the first time, genetic loci for maternal incubation behaviour  that is derived from crossing the SLK breed which shows the behaviour and WL breed which does not. The trait was confirmed to be dominant with 97 % incubation behaviour in the F1 generation. The QTL on chromosomes 5, 13, 18, 19 and in linkage group E22C19W28 suggest that the alleles inherited from the WL promote incubation behaviour or that the performance of the heterozygotes exceeds that of the homozygotes. We believe this could be due to differences in the hen’s fertility which increase the chance that the hen demonstrates incubation behaviour over the period of the test. Analysis of the QTL on chromosomes 1 and 8 for early incubation behaviour trait, which is unlikely to be affected by fertility, shows that they are inherited as would be predicted with the SLK allele promoting early incubation behaviour. The coincidence of a number of the QTL with aspects of the thyrotrophic axis suggests that the thyroid hormone system may be critical for loss of incubation behaviour and/or improved egg laying.

## References

[1] Sharp PJ. Broodiness and broody control. In: Hocking PM, editor. Biology of breeding poultry. Poultry Science Symposium Series. Wallingford: CABI; 2009. p. 181–205.

[2] Romanov MN (2001). Genetics of broodiness in poultry—a review. Asian Aust J Anim Sci..

[3] Romanov MN, Talbot RT, Wilson PW, Sharp PJ (1999). Inheritance of broodiness in the domestic fowl. Br Poult Sci.

[4] Romanov MN, Talbot RT, Wilson PW, Sharp PJ (2002). Genetic control of incubation behavior in the domestic hen. Poult Sci.

[5] Hutt F (1949). Genetics of the fowl.

[6] Squires EJ (2010). Effects on animal behaviour, health and welfare. Applied animal endocrinology.

[7] Collias NE, Collias EC (1967). A field study of red jungle fowl in north-central India. Condor..

[8] Banner E (1916). The history and development of artificial incubation.

[9] Saeki Y, Inoue Y (1979). Body growth, egg production, broodiness, age at first egg and egg size in Red Jungle fowls, and an attempt at their genetic analyses by the reciprocal crossing with White Leghorns. Jpn Poult Sci..

[10] Zhou M, Lei M, Rao Y, Nie Q, Zeng H, Xia M (2008). Polymorphisms of *vasoactive intestinal peptide receptor*-*1* gene and their genetic effects on broodiness in chickens. Poult Sci.

[11] Liang Y, Cui JX, Yang GF, Leung FCC, Zhang XQ (2006). Polymorphisms of 5′ flanking region of chicken *prolactin* gene. Domest Anim Endocrinol.

[12] van Wulfften Palthe A. C.S.Th van Gink’s Poultry Paintings 1890–1968. Beekbergen: Dutch Branch of the World’s Poultry Science Association (WPSA); 1992.

[13] Punnett RC, Bailey PG (1920). Genetic studies in poultry. II. Inheritance of egg-colour and broodiness. J Genet..

[14] Hays F (1940). Inheritance of broodiness in Rhode Island Reds. Mass Agric Exp Stn Bull.

[15] Saeki Y (1957). Inheritance of broodiness in Japanese Nagoya fowl, with special reference to sex-linkage and notice in breeding practice. Poult Sci.

[16] Kitalyi AJ. Village chicken production systems in developing countries: What does the future hold? World Anim Rev. 1997;89:48–53 .http://www.fao.org/docrep/W6437T/w6437t07.

[17] Dunn IC, Paton IR, Clelland AK, Sebastian S, Johnson EJ, McTeir L (2011). The chicken *polydactyly* (*Po*) locus causes allelic imbalance and ectopic expression of Shh during limb development. Dev Dyn.

[18] Dunn IC, Joseph NT, Bain M, Edmond A, Wilson PW, Milona P (2009). Polymorphisms in eggshell organic matrix genes are associated with eggshell quality measurements in pedigree Rhode Island Red hens. Anim Genet.

[19] Groenen MAM, Wahlberg P, Foglio M, Cheng HH, Megens HJ, Crooijmans R (2009). A high-density SNP-based linkage map of the chicken genome reveals sequence features correlated with recombination rate. Genome Res.

[20] Law AS, Archibald AL (2000). Farm animal genome databases. Brief Bioinform..

[21] Paterson T, Law A (2011). GENOTYPECHECKER: an interactive tool for checking the inheritance consistency of genotyped pedigrees. Anim Genet.

[22] Haley CS, Knott SA, Elsen JM (1994). Mapping quantitative trait loci in crosses between outbred lines using least squares. Genetics.

[23] Seaton G, Hernandez J, Grunchec J-A, White I, Allen J, De Koning DJ, et al. GridQTL: a grid portal for QTL mapping of compute intensive datasets. In Proceedings of the 8th World Congress on Genetics Applied to Livestock Production: 13–18 August 2006; Belo Horizonte; 2006.

[24] Seaton G, Haley CS, Knott SA, Kearsey M, Visscher PM (2002). QTL Express: mapping quantitative trait loci in simple and complex pedigrees. Bioinformatics.

[25] Churchill GA, Doerge RW (1994). Empirical threshold values for quantitative trait mapping. Genetics.

[26] Lander ES, Botstein D (1989). Mapping mendelian factors underlying quantitative traits using RFLP linkage maps. Genetics.

[27] Visscher PM, Thompson R, Haley CS (1996). Confidence intervals in QTL mapping by bootstrapping. Genetics.

[28] Kruglyak L, Lander ES (1995). A nonparametric approach for mapping quantitative trait loci. Genetics.

[29] Rubin CJ, Zody MC, Eriksson J, Meadows JRS, Sherwood E, Webster MT (2010). Whole-genome resequencing reveals loci under selection during chicken domestication. Nature.

[30] Fan WL, Ng CS, Chen CF, Lu MYJ, Chen YH, Liu CJ (2013). Genome-wide patterns of genetic variation in two domestic chickens. Genome Biol Evol..

[31] Flink LG, Allen R, Barnett R, Malmstrom H, Peters J, Eriksson J (2014). Establishing the validity of domestication genes using DNA from ancient chickens. Proc Natl Acad Sci USA.

[32] Nakao N, Ono H, Yamamura T, Anraku T, Takagi T, Higashi K (2008). Thyrotrophin in the pars tuberalis triggers photoperiodic response. Nature.

[33] Hu Z-L, Reecy JM (2007). Animal QTLdb: beyond a repository. Mamm Genome.

[34] Zhou H, Evock-Clover CM, McMurtry JP, Ashwell CM, Lamont SJ (2007). Genome-wide linkage analysis to identify chromosomal regions affecting phenotypic traits in the chicken. IV. Metabolic traits. Poult Sci..

[35] Nadaf J, Pitel F, Gilbert H, Duclos MJ, Vignoles F, Beaumont C (2009). QTL for several metabolic traits map to loci controlling growth and body composition in an F(2) intercross between high- and low-growth chicken lines. Physiol Genomics.

[36] Tuiskula-Haavisto M, De Koning DJ, Honkatukia M, Schulman NF, Maki-Tanila A, Vilkki J (2004). Quantitative trait loci with parent-of-origin effects in chicken. Genet Res.

[37] Podisi BK, Knott SA, Dunn IC, Law AS, Burt DW, Hocking PM (2011). Overlap of quantitative trait loci for early growth rate, and for body weight and age at onset of sexual maturity in chickens. Reproduction.

[38] Hansen C, Yi NJ, Zhang YM, Xu SZ, Gavora J, Cheng HH (2005). Identification of QTL for production traits in chickens. Anim Biotechnol..

[39] Tuiskula-Haavisto M, Honkatukia M, Vilkki J, de Koning DJ, Schulman NF, Maki-Tanila A (2002). Mapping of quantitative trait loci affecting quality and production traits in egg layers. Poult Sci.

[40] Atzmon G, Blum S, Feldman M, Lavi U, Hillel J (2007). Detection of agriculturally important QTLs in chickens and analysis of the factors affecting genotyping strategy. Cytogenet Genome Res..

[41] Dawson A, Talbot RT, Dunn IC, Sharp PJ (2002). Changes in basal hypothalamic chicken gonadotropin-releasing hormone-I and vasoactive intestinal polypeptide associated with a photo-induced cycle in gonadal maturation and prolactin secretion in intact and thyroidectomized starlings (*Sturnus vulgaris*). J Neuroendocrinol.

[42] Hertwig P, Schwarz E (1934). Brutigkeit und Ovarhormon. Arch Geflugelk..

